# The validity of the labelling index in tumour studies.

**DOI:** 10.1038/bjc.1985.3

**Published:** 1985-01

**Authors:** E. Hamilton, J. Dobbin

## Abstract

The distribution of labelled cells through 5 different mouse tumours was measured after a single injection of [3H]-thymidine [( 3H]-TdR) or [3H]-deoxyuridine [( 3H]-UdR). All the tumours had areas where the percentage of labelled cells (the labelling index, LI) was high and areas where the LI was very low. The total area with a low LI was greater after [3H]-TdR than after [3H]-UdR injection in all 5 tumours. In one of the tumours, carcinoma NT, repeated injections of [3H]-UdR at 2 h intervals caused the areas of high LI to spread, eliminating all areas of low LI in many specimens. When 5-fluorodeoxyuridine (FUdR) was injected, to block de novo DNA synthesis in carcinoma NT, [3H]-TdR was incorporated by many more cells. The LI was increased throughout the tumour and no area had a LI below 20% after FUdR plus [3H]-TdR. After flash-labelling with [3H]-TdR alone, nearly half the tumour had a LI below 20%. We conclude that the labelling seen after FUdR plus [3H]-TdR represented the true distribution of S phase cells in carcinoma NT. Routine flash-labelling with [3H]-TdR or [3H]-UdR left nearly half the S phase cells unlabelled and gave an erroneously low value for the proportion of DNA synthesising cells in the tumour. The results suggest that many tumour cells have very large endogenous nucleotide pools which cannot be flooded by a single injection, even of [3H]-UdR.


*Br. J. Cancer* (1985), **51**, 15–21

### The validity of the labelling index in tumour studies

E. Hamilton* and J. Dobbin

*Department of Oncology, Middlesex Hospital Medical School, London W1, UK.*

**Summary** The distribution of labelled cells through 5 different mouse tumours was measured after a single injection of [$^3$H]-thymidine ([$^3$H]-TdR) or [$^3$H]-deoxyuridine ([$^3$H]-UdR). All the tumours had areas where the percentage of labelled cells (the labelling index, LI) was high and areas where the LI was very low. The total area with a low LI was greater after [$^3$H]-TdR than after [$^3$H]-UdR injection in all 5 tumours.

In one of the tumours, carcinoma NT, repeated injections of [$^3$H]-UdR at 2 h intervals caused the areas of high LI to spread, eliminating all areas of low LI in many specimens. When 5-fluorodeoxyuridine (FUdR) was injected, to block *de novo* DNA synthesis in carcinoma NT, [$^3$H]-TdR was incorporated by many more cells. The LI was increased throughout the tumour and no area had a LI below 20% after FUdR plus [$^3$H]-TdR. After flash-labelling with [$^3$H]-TdR alone, nearly half the tumour had a LI below 20%. We conclude that the labelling seen after FUdR plus [$^3$H]-TdR represented the true distribution of S phase cells in carcinoma NT. Routine flash-labelling with [$^3$H]-TdR or [$^3$H]-UdR left nearly half the S phase cells unlabelled and gave an erroneously low value for the proportion of DNA synthesising cells in the tumour. The results suggest that many tumour cells have very large endogenous nucleotide pools which cannot be flooded by a single injection, even of [$^3$H]-UdR.

Cells flash-labelled with [$^3$H]-thymidine ([$^3$H]-TdR) are unevenly distributed through tumours *in vivo*. Areas with few labelled cells are generally thought to be nonproliferating. However, the uneven distribution of labelled cells might also arise from the pattern of [$^3$H]-TdR diffusion through the tumour (Kligermann *et al.*, 1962). Tannock (1968) found that the median grain count over labelled cells in tumour cords fell with the distance from the capillary, while the length of DNA synthesis ($T_S$) remained constant. *In vitro*, where label was freely available, nuclear grain count was proportional to $1/T_S$ (Dörmer *et al.*, 1975), so Tannock's (1968) results may suggest that the availability of [$^3$H]-TdR decreased across tumour cords.

Several studies in which DNA content, measured by flow cytometry, was compared with [$^3$H]-TdR labelling have shown that not all S phase cells incorporated the label. In the mouse epidermal basal layer, only 80% of cells with mid S phase DNA content incorporated [$^3$H]-TdR (Clausen *et al.*, 1980). In EMT-6 multicellular spheroids the percentage of labelled S phase cells fell from 100% at the surface to 23% at a depth of 150 $\mu$m (Freyer & Sutherland, 1980). Thus unlabelled S phase cells have been found in slowly cycling populations and where nutrients are limited. The latter condition is characteristic of solid tumours and there is also evidence that many cells in a solid tumour cycle slowly (Hamilton & Dobbin, 1983*a*, *b*). The apparently viable areas of tumour tissue lacking labelled cells which have been reported in various tumours (Rockwell *et al.*, 1972; Hirst *et al.*, 1982) may therefore contain unlabelled cycling cells rather than "resting" cells.

We have shown that in the periphery of the mouse carcinoma NT [$^3$H]-TdR was incorporated by many fewer cells than was [$^3$H]-deoxyuridine ([$^3$H]-UdR) (Hamilton & Dobbin, 1982). In the present study we have used various methods to increase the penetration of both [$^3$H]-TdR and [$^3$H]-UdR into carcinoma NT. These results are compared with the labelling produced by a single injection of [$^3$H]-TdR or [$^3$H]-UdR in carcinoma NT and in 4 other tumours of different histological types.

### Materials and methods

*Carcinoma NT*, an undifferentiated adenocarcinoma of spontaneous origin, was serially transplanted in isogenic CBA mice. A 1 mm cube of tumour tissue was implanted subcutaneously on the dorsum of 3–4 month old ♂ mice. Tumours with a volume doubling time ($T_D$) of 3–6 days were studied when they reached a diameter of 7–10 mm, equivalent to a weight of 180–520 mg.

For flash labelling 30 $\mu$Ci 6[$^3$H]-TdR, specific activity 5 Ci mM$^{-1}$ (Amersham International PLC) or an equimolar quantity of 6[$^3$H]-UdR, 50 $\mu$Ci of specific activity 15–20 Ci mM$^{-1}$, was injected i.p. Groups of 6 animals were killed 45 min later and

Correspondence: E. Hamilton.

Received 21 February 1984; and in revised form, 26 September 1984.

*Present address: ICI Pharmaceuticals Division, Mereside, Alderley Park, Macclesfield, Cheshire SK10 4TG, U.K.

© The Macmillan Press Ltd., 1985

the tumours were fixed in Carnoy's solution overnight at 4°C.

For repeated labelling 25 $\mu$Ci of [$^3$H]-UdR was injected every 2 h, for a total of 4 injections. A group of 6 animals was killed 45 min after each injection.

5-Fluorodeoxyuridine (FUdR) (Sigma London Chemical Co.) was dissolved in saline and mice were injected with 0.2, 0.8 or 1.6 mg of the drug. This was equivalent to 0.8, 3.2 and $6.5 \times 10^{-5}$ moles of FUdR respectively. [$^3$H]-TdR (50 $\mu$Ci) was injected 20–60 min after the FUdR and the tumours were sampled 45 min later.

*GSF Tumours.* Four tumour lines maintained in C3H mice at the Institut für Biologie, GSF, Munich were used in these studies. Adenoacanthoma *AT 17* was well differentiated with a $T_D$ of 8 days at 100 mg and 18 days at 500 mg size. Adenocarcinoma *284* was moderately differentiated with a $T_D$ of 2.0 and 4.5 days at 100 and 500 mg respectively. Mammary carcinoma *AT 7* had an undifferentiated structure and a $T_D$ of 2.5 and 5.5 days at 100 and 500 mg. Fibrosarcoma *SSK2* was also undifferentiated and grew most rapidly with a $T_D$ of 1.5 days at 100 mg and 2.7 days at 500 mg. Tumours AT17 and AT7 were radiation induced and SSK2 was cloned from a methyl-cholanthrene induced tumour. All 3 were transplanted in the strain of origin. Tumour 284 arose spontaneously in a syngeneic strain.

For each tumour line 2 small tumours (close to 100 mg) and 2 large tumours (up to 500 mg) were labelled with an injection of 2 $\mu$Ci g$^{-1}$ body wt ($\sim$60 $\mu$Ci total) [$^3$H]-TdR or [$^3$H]-UdR, at GSF by Dr J. Kummermehr. The tumours were fixed in alcoholic formalin 1 h after the injection and were transported to the Middlesex Hospital Medical School.

All tumours were embedded in wax, sectioned at 4 $\mu$m and dipped in Ilford K5 emulsion diluted 1:1 with water. The slides were exposed for 3 weeks, developed in Kodak D19 and stained with H and E. The background was no more than 3 grains per nucleus and cells with 5 or more grains were regarded as labelled.

The labelling index (LI) calculated from labelled/total cells × 100%, was counted in 25 randomly distributed fields in each tumour. A Chalkley point array was placed over a section at ×25 magnification and at each of the 25 points a 180 × 180 $\mu$m field of cells was counted under ×400 magnification. Where points lay over necrotic areas, no field was counted. The LI values from each individual field in all the tumours in a group were plotted in a histogram. Differences between histograms were analysed by means of the Kolmogorov–Smirnov (K–S) 2-tailed test (Young, 1977). An overall LI value was calculated for each tumour by adding all the counts of labelled and unlabelled cells. This gave a total of 3,500–5.000 cells per tumour in carcinoma NT, 6,200–8,200 in 284, 6,500–7,300 in SSK2, 6,600–7,900 in AT17 and 2,500–4,100 cells per tumour in AT7.

In some tumours the LI was also measured non-randomly. An eyepiece grid was placed over areas of heavy labelling and all cells in the 180 × 180 $\mu$m field were counted. This was repeated until at least 2,000 cells had been counted.

### Results

Figure 1 shows distribution of LI values in individual random fields in carcinoma NT flash-labelled with [$^3$H]-TdR or [$^3$H]-UdR. The LI ranged from <5% in some areas to >50% in others.

The figure demonstrates that more fields had low LI values in tumours labelled with [$^3$H]-TdR than in those labelled with [$^3$H]-UdR. However, the K–S test showed the Figure 1 histograms were not significantly different, nor were the overall mean LI values for the [$^3$H]-TdR and [$^3$H]-UdR labelled tumours, as shown in Table I. In order to quantify the difference between the [$^3$H]-TdR and [$^3$H]-

**Figure 1** The distribution of LI values in individual random fields in carcinoma NT flash-labelled with [$^3$H]-TdR or [$^3$H]-UdR. Figures in brackets indicate number of fields counted.

**Table I** Percentage labelling index (LI) in random fields in 5 tumour lines after a single injection of [$^3$H]-TdR or [$^3$H]-UdR.

| | | | [$^3H$]-*thymidine* | | [$^3H$]-*deoxyuridine* | |
|---|---|---|---|---|---|---|
| *Tumour* | *Range of LI in fields* | *Limit of "low" LI*[a] | *Overall mean LI* ($\pm$*se*) | *Proportion of fields below limit* | *Overall mean LI* ($\pm$*s.e.*) | *Proportion of fields below limit* |
| Carcinoma NT | 1–55 | 20 | 26.7 ± 1.2 | 0.44 | 27.3 ± 1.4 | 0.33 |
| Adenocarcinoma 284 | 12–61 | 32 | 37.9 ± 2.9 | 0.33 | 41.2 ± 2.6 | 0.24 |
| Fibrosarcoma SSK2 | 24–62 | 35 | 42.2 ± 2.0 | 0.20 | 44.0 ± 1.5 | 0.04[b] |
| Adenocanthoma AT17 | 4–44 | 18 | 20.9 ± 2.9 | 0.44 | 26.0 ± 2.8 | 0.23[c] |
| Mammary carcinoma AT7 | 9–58 | 27 | 29.3 ± 3.0 | 0.51 | 37.3 ± 0.7[d] | 0.11[c] |

[a]Limit = 0.85 of median LI in range. [$^3$H]-TdR and [$^3$H]-UdR histograms different. [b]$P < 0.05$. [c]$P < 0.005$.
[d][$^3$H]-TdR and [$^3$H]-UdR mean LI different, $P < 0.02$.

UdR labelling patterns, a limit was set below which fields had a "low" LI. Subsequent experiments produced many tumours in which no field had an LI below 20% (Tables II and III), so this value was used as the limit for "low" LI in Figure 1. After [$^3$H]-TdR flash-labelling 0.44 of the fields were below this limit, compared with 0.33 of fields in [$^3$H]-UdR labelled carcinoma NT (Table I). The difference between the two precursors was also demonstrated by non-random counts of the LI, which gave mean values of 42.9 ± 0.9% for [$^3$H]-TdR and 48.2 ± 1.1% for [$^3$H]-UdR labelled tumours.

The distribution of [$^3$H]-TdR and [$^3$H]-UdR labelling in the 4 tumours from GSF is also given in Table I and representative histograms, from AT17, are shown in Figure 2. In carcinoma NT the "low" LI limit of 20% was 0.85 of the median LI found in individual fields (Figure 1). "Low" LI limits were set by the same criterion for the other 4 tumours. The range of LI values in individual fields and the "low" LI limit derived from it, was different in each tumour line (Table I). However, in all the tumours fewer fields had a "low" LI after [$^3$H]-UdR than after [$^3$H]-TdR flash-labelling. The K–S test showed that in all the tumours but 284 the histograms of individual LI values in [$^3$H]-TdR and [$^3$H]-UdR labelled tumours (Figure 2) were significantly different. However, the overall mean LI with [$^3$H]-TdR was significantly below that with [$^3$H]-UdR in only one tumour, AT7 (Table I).

Table II shows how the labelling pattern in carcinoma NT changed with repeated injections of [$^3$H]-UdR. Among each group of 6 tumours sampled after 2, 3 or 4 injections, two patterns of labelling with significantly different mean LI values were found. In tumours with one labelling pattern

**Figure 2** The distribution of LI values in individual random fields in adenocanthoma AT17 flash-labelled with [$^3$H]-TdR or [$^3$H]-UdR. Total number of fields shown in brackets.

the LI in individual fields ranged from <10% to >60% (Table II). Tumours with the second pattern of labelling had no area with a LI below 20% (the "low" LI limit), as shown in Figure 3. The labelling pattern in Figure 3 is significantly different from that found after a single injection of [$^3$H]-

**Table II** The effect of repeated injections of [$^3$H]-UdR on the pattern of labelling in carcinoma NT.

| Injections of [$^3H$]-UdR | Overall mean LI for all tumours ± s.e. | Tumours with "low" LI fields: Number | Overall mean LI (%) | Range of LI in fields (%) | Tumours with no "low" LI fields: Number | Overall mean LI (%) | Range of LI in fields (%) |
|---|---|---|---|---|---|---|---|
| 1 | 27.3±1.4 | 6 | 27.3±1.4 | 1–48 | 0 | — | — |
| 2 | 33.4±1.6 | 5 | 32.3±1.4 | 10–60 | 1 | 39.0 | 27–63 |
| 3 | 34.3±2.5 | 4 | 31.4±2.6 | 12–68 | 2 | 40.3±1.9 | 22–68 |
| 4 | 44.6±2.4 | 2 | 38.1±2.8 | 4–68 | 4 | 47.9±1.7 | 23–72 |

**Figure 3** The distribution of LI values in individual random fields in carcinoma NT injected with 25 $\mu$Ci [$^3$H]-UdR at 2 hourly intervals. Upper panel, 3 tumours given 2 or 3 injections, lower panel, 4 tumours given 4 injections.

UdR (Figure 1). The proportion of tumours without "low" LI fields increased with the number of injections (Table II). The overall mean LI values for tumours with both labelling patterns increased after each injection of [$^3$H]-UdR.

In tumours treatedwith 0.2 mg FUdR followed by [$^3$H]-TdR, 3 patterns of labelling were seen (Table III). In 6 tumours, given [$^3$H]-TdR 20 or 40 min after FUdR, a maximum of 1 out of 25 random fields had a LI below 20% (Group 1). In another 6 tumours, given [$^3$H]-TdR 20, 40 or 60 min after FUdR, there was at most 1 field with a LI below 10% (Group 2). The pattern of labelling in both these groups was significantly different from that produced by [$^3$H]-TdR alone (Figure 1). The third group, of 3 tumours, had many fields with a LI below 10% and a labelling pattern similar to that found after [$^3$H]-TdR alone. These tumours were injected with [$^3$H]-TdR 40 or 60 min after FUdR. All 15 tumours had similar mitotic indices, proportion of necrotic tissue and $T_D$.

The dose of FUdR was increased to 0.8 or 1.6 mg per mouse and [$^3$H]-TdR was injected 30 min later, in an attempt to eliminate low LI fields in all tumours. However, two patterns of labelling were found after both these doses. In half the tumours, (Group 4) no field had a LI below 25%, while in the rest of the tumours (Group 5) LI values below 20% were found. The patterns of labelling in these 2 groups of tumours is shown in Figure 4. Both histograms were significantly different from that

**Table III** The effect of FUdR on labelling patterns in carcinoma NT

| Dose of FUdR | Time, FUdR to [$^3H$]-TdR (min) | Group | Number of tumours | Overall mean LI (%) | Range of LI in fields (%) |
|---|---|---|---|---|---|
| 0.2 mg | 20, 40 | 1 | 6 | 45.2±1.2 | 15–65 |
| | 20, 40, 60 | 2 | 6 | 32.0±1.9 | 5–56 |
| | 40, 60 | 3 | 3 | 27.2±2.5 | 1–48 |
| 0.8 or 1.6 mg | 30 | 4 | 5 | 46.9±1.5 | 27–70 |
| | 30 | 5 | 5 | 36.6±2.0 | 6–66 |

**Figure 4** The distribution of LI values in individual random fields in carcinoma NT given 0.8 or 1.6 mg FUdR 30 min before [$^3$H]-TdT. (a) tumours with no LI below 25%, (b) tumours with low LI values.

produced by [$^3$H]-TdR alone (Figure 1). The histograms and overall mean LI values for Group 1 (0.2 mg) and Group 4 (0.8, 1.6 mg) were not significantly different (Table III). The maximum LI values found in groups 1, 4 and 5 were higher than those measured after [$^3$H]-TdR or [$^3$H]-UdR alone (Table I).

In carcinoma NT flash-labelled with [$^3$H]-TdR or [$^3$H]-UdR the number of grains per labelled nucleus ranged from 5 to >80. It was not possible to count individual grains when >80 lay above a nucleus. In areas of high LI in tumours flash-labelled with [$^3$H]-UdR, half the labelled nuclei had >80 grains above them (Hamilton & Dobbin, 1983*b*). After FUdR treatment most of the labelled nuclei in areas of high LI had >80 grains. In areas where the LI was lower however, the grain count per nucleus was more varied. Nuclei with >80 grains were often found close to the necrosis, but there were patches of tumour parenchyma where no cell had >20 grains.

### Discussion

The technique of counting large numbers of randomly placed fields gives the most representative sample of an entire tumour. Because the fields are randomly placed, the total number in any category shows the area of the tumour occupied by that category of tissue. The results in Table I show that in 5 tumour lines of differing histology the area with a low LI was larger after [$^3$H]-TdR than after [$^3$H]-UdR flash labelling. The difference between [$^3$H]-UdR and [$^3$H]-TdR labelling patterns was significant in 3 of the tumour lines. However, the overall mean LI, averaged over the entire tumour, was significantly lower after [$^3$H]-TdR than after [$^3$H]-UdR flash-labelling in only 1 tumour line.

In carcinoma NT a third of the tumour area had a LI below 20% after a single injection of [$^3$H]-UdR (Table I, Figure 1). In many of the tumours given 2 to 4 injections of [$^3$H]-UdR at 2 hourly intervals, no area with a LI below 20% was found (Table II, Figure 3). An injection of FUdR given before ($^3$H]-TdR also reduced the area of low LI (Table III) and in many FUdR treated tumours there was no area with a LI below 20% (Figure 4). FUdR treatment increased the overall mean LI to 46%, from 27% in flash-labelled tumours.

The pattern of labelling in the tumours with the least area of low LI after 0.2, 0.8 or 1.6 mg FUdR (Table III, Groups 1 and 4) was not significantly different. This may be the best labelling which can be obtained in carcinoma NT and may represent the true distribution of DNA synthesising cells through the tumour. Thus 46% of the viable cells were actively synthesising DNA (Table III) and in no part of the tumour were fewer than a quarter of the cells in S phase (Figure 4a). Tumours with fewer labelled cells than those shown in Figure 4a either contained unlabelled cells in S phase, or had many fewer DNA synthesising cells. The latter explanation is unlikely since the mean mitotic indices for the groups of tumours with different labelling patterns were the same. The histology and $T_D$ of all the tumours treated with FUdR were also very similar, suggesting that they all had similar growth kinetics. The presence of areas with a LI below 20% and an overall mean LI below 46% in carcinoma NT therefore indicates that not all S phase cells in the tumour were labelled.

In some tumours given repeated injections of [$^3$H]-UdR, shown in Figure 3, the range of LI values in individual fields was the same as that in Figure 4a. However, the overall mean LI in the repeatedly labelled tumours was significantly lower than that after FUdR plus [$^3$H]-TdR (Table II, III). Therefore, even in these repeatedly labelled tumours some S phase cells remained unlabelled. In carcinoma NT cells entered S phase at a rate of 2.3% $h^{-1}$ (Hamilton & Dobbin, 1983*a*, *b*). This entry to S phase contributed to the increase in overall LI with repeated injections (Table II), but was insufficient to account for the loss of all areas with a LI below 20%, even after 4 injections. The loss of these areas in an increasing number of tumours after each injection suggests that large nucleotide pools were gradually flooded by the label.

The effect of FUdR on the tumours also demonstrates the presence of large nucleotide pools. FUdR binds to thymidylate synthetase and blocks *de novo* synthesis of thymidine monophosphate from deoxyuridine monophosphate (Myers *et al.*, 1975). Cells can maintain a normal rate of DNA synthesis in the presence of FUdR by incorporating thymidine through the alternative, salvage pathway (Dörmer *et al.*, 1975). There is no suggestion in the literature that FUdR, an inhibitor of DNA synthesis, stimulates "resting" cells into cycle. Therefore it is likely that all the cells which incorporated [$^3$H]-TdR after FUdR treatment were actively synthesising DNA at the time of the experiment (Meyer & Facher, 1977). The results in Table II show 20 to 40 min to be the optimum interval between FUdR and [$^3$H]-TdR injection. This suggests that synthesis had to proceed by the salvage pathway for some time before the nucleotide pools were reduced to a level where they could be flooded by label.

Half the tumours treated with 0.8 or 1.6 mg FUdR retained areas where the LI was below 20%. Similarly 2 of the 6 tumours given 4 injections of [$^3$H]-UdR had areas where the LI was under 20%. This variation between tumours in the pattern of labelling suggests that not only were the endogenous nucleotide pools large, but their size and constituents varied between tumours.

The binding of FUdR to thymidylate synthetase is inhibited by deoxyuridine monophosphate and slowed by deoxyuridine and other nucleotides (Lockskin & Danenberg, 1981). Variations in the intracellular concentrations of these molecules would therefore alter the degree of inhibition of thymidylate synthetase by FUdR. This may be why a given dose of FUdR did not always cause every S phase cell to be labelled (Figure 4, Table III). We did not increase the FUdR dose further because of the inhibitory effect of the drug. A dose of 120 mg kg$^{-1}$ 5-FU (equivalent to $2.8 \times 10^{-5}$ mol mouse$^{-1}$) inhibited DNA synthesis for 24 h in solid tumours in mice and rats (Klubes *et al.*, 1978).

An alternative explanation for the variable labelling patterns after FUdR or repeated [$^3$H]-UdR injections is that the areas of low LI were served by vessels closed to the circulation between the time of injection and death (Hirst *et al.*, 1982). Tannock & Steel (1969) showed that some small vessels in a rat tumour contained static blood for a period of 10 min. However, the 6 tumours which retained areas of low LI after 3 or 4 injections of [$^3$H]-UdR (Table II) had been exposed to label, intermittently, over a period of 5 or 7 h. If the areas of low LI in these tumours were caused by vascular stasis, the vessels must have been closed for at least 5 (or 7) h. Cells cut off from the circulation for that length of time would probably become necrotic, rather than remaining viable but unlabelled. Therefore, in the repeatedly labelled tumours and probably also in other tumours, areas of low LI must have been due to an effect other than vascular stasis.

[$^3$H]-TdR was incorporated by at least a quarter of the cells throughout FUdR treated tumours. This suggests that the large areas of low LI in tumours flash-labelled with [$^3$H]-TdR may not have been caused by poor distribution of the label. The smaller area of low LI after [$^3$H]-UdR flash-labelling again demonstrates that different nucleotides varied in abundance in the endogenous pools.

In the 4 tumours labelled at GSF, more moles of [$^3$H]-TdR than of [$^3$H]-UdR were injected, because of the different specific activities of the 2 nucleotides. [$^3$H]-TdR should therefore have flooded endogenous pools to a greater extent than [$^3$H]-UdR, but in all 4 tumours the area of low LI was larger with [$^3$H]-TdR (Table I, Figure 2). This demonstrates that these tumours also had large endogenous nucleotide pools and suggests that they too, like carcinoma NT, contained S phase cells unlabelled by a single injection. This may also be the explanation for the area of unlabelled viable tissue reported in other tumours labelled with [$^3$H]-TdR (Rockwell *et al.*, 1972; Hirst *et al.*, 1982; Camplejohn, personal communication).

Histograms of the LI values for individual fields showed significant differences between [$^3$H]-TdR and [$^3$H]-UdR incorporation in 3 GSF tumours (Table I). However, the overall mean LI values calculated from the same counts were only significantly different in one tumour, AT7. This is because the difference between [$^3$H]-TdR and [$^3$H]-UdR was in the area of low LI, while regions of high LI contributed more to the overall mean. Counting procedures whereby large numbers of fields are combined to give an overall LI therefore mask poor labelling of S phase cells.

In carcinoma NT where 46% of cells were in S phase (Table III), the randomly counted mean LI after flash labelling was only 27% (Table I). This LI therefore underestimated the proportion of DNA synthesising cells in the tumour by about half. Non-random counts of the flash-labelled tumours, made only in areas of heavy labelling, gave LI values of 43% ([$^3$H]-TdR) and 48% ([$^3$H]-UdR), not significantly different from the true proportion of S phase cells (Table III). The correspondence between these values may be fortuitous, but it indicates that counts which exclude areas of low LI may give the best estimate of S phase cells in flash labelled tumours.

In tumours where labelling is inhomogenous after a single injection, the possibility of incomplete labelling of S phase cells, as shown above, must be considered. Sparsely labelled areas should not be assumed to contain non-cycling cells without further studies, for example with FUdR.

We would like to thank Dr J. Kummermehr of GSF, Neuherberg, for providing labelled specimens of 4 tumour lines. Thanks also go to Prof R.J. Berry, for his advice and assistance. This work was supported by the Frances and Augustus Newman Foundation.

### References

CLAUSEN, O.P.F., THORUD, E. & BOLUND, L. (1980).. DNA synthesis in mouse epidermis. Labelled and unlabelled basal cells in S phase after administration of tritiated thymidine. *Vichows Archiv B*, **34**, 1.

DÖRMER, P., BRINKMANN, W. & DAHR, P. (1972). Thymidylat-de novo-synthese, salvage pathway und DNS-synthese während der differenzierung von erythroblasten. *Blut*, **25**, 185.

DÖRMER, P., BRINKMANN, W., BORN, R. & STEEL, G.G. (1975). Rate and time of DNA synthesis of individual chinese hamster cells. *Cell Tissue Kinet*, **8**, 399.

FREYER, J.P. & SUTHERLAND, R.M. (1980). Selective dissociation and characterization of cells from different regions of multicell tumor spheroids. *Cancer Res.*, **40**, 3956.

HAMILTON, E. & DOBBIN, J. (1982). $^3$H-thymidine labels less than half of the DNA-synthesizing cells in the mouse tumour, carcinoma NT. *Cell Tissue Kinet.*, **15**, 405.

HAMILTON, E. & DOBBIN, J. (1983*a*). The percentage labelled mitoses technique shows the mean cell cycle time to be half its true value in carcinoma NT. I $^3$H-thymidine and vincristine studies. *Cell Tissue Kinet.*, **16**, 473.

HAMILTON, E. & DOBBIN, J. (1983*b*). The percentage labelled mitoses technique shows the mean cell cycle time to be half its true value in carcinoma NT. II $^3$H-deoxyuridine studies. *Cell Tissue Kinet.*, **16**, 483.

HIRST, D.G., DENEKAMP, J. & HOBSON, B. (1982). Proliferation kinetics of endothelial and tumour cells in 3 mouse mammary carcinomas. *Cell Tissue Kinet.*, **15**, 251.

KLIGERMAN, M.M., HEIDENREICH, W.F. & GREENE, S. (1962). Distribution of tritiated thymidine about a capillary sinusoid in a transplanted mouse tumour. *Nature*, **196**, 282.

KLUBES, P., CONNELLY, K., CERNA, I. & MANDEL, H.G. (1978). Effects of 5-fluorouracil on 5-fluorodeoxyuridine 5$^1$-monophosphate and 2-deoxyuridine 5$^1$-monophosphate pools and DNA synthesis in solid mouse L1210 and rat Walker 256 tumors. *Cancer Res.*, **38**, 2325.

LOCKSHIN, A. & DANENBERG, P.V. (1981). Biochemical factors affecting the tightness of 5-fluorodeoxyuridylate binding to human thymidylate synthetase. *Biochem. Pharmacol.*, **30**, 247.

MEYER, J.S. & FACHER, R. (1977). Thymidine labelling index of human breast carcinoma. *Cancer*, **39**, 2524.

MYERS, C.E., YOUNG, R.C. & CHABNER, B.A. (1975). Biochemical determinants of 5-fluorouracil response *in vivo*. *J. Clin. Invest.*, **56**, 1231.

ROCKWELL, S.C., KALLMAN, R.J. & FAJARDO, L.F. (1972). Characteristics of a serially transplanted mouse mammary tumour and its tissue-culture adapted derivative. *J. Natl. Cancer Inst.*, **49**, 735.

TANNOCK, I.F. (1968). The relation between cell proliferation and the vascular system in a transplanted mouse mammary tumour. *Br. J. Cancer*, **22**, 258.

TANNOCK, I.F. & STEEL, G.G. (1969). Quantitative techniques for study of the anatomy and function of small blood vessels in tumours. *J. Natl. Cancer Inst.*, **42**, 771.

YOUNG, I.T. (1977). Proof without prejudice: use of the Kolmogorov–Smirnov test for the analysis of histograms from flow systems and other sources. *J. Histochem. Cytochem.*, **25**, 935.


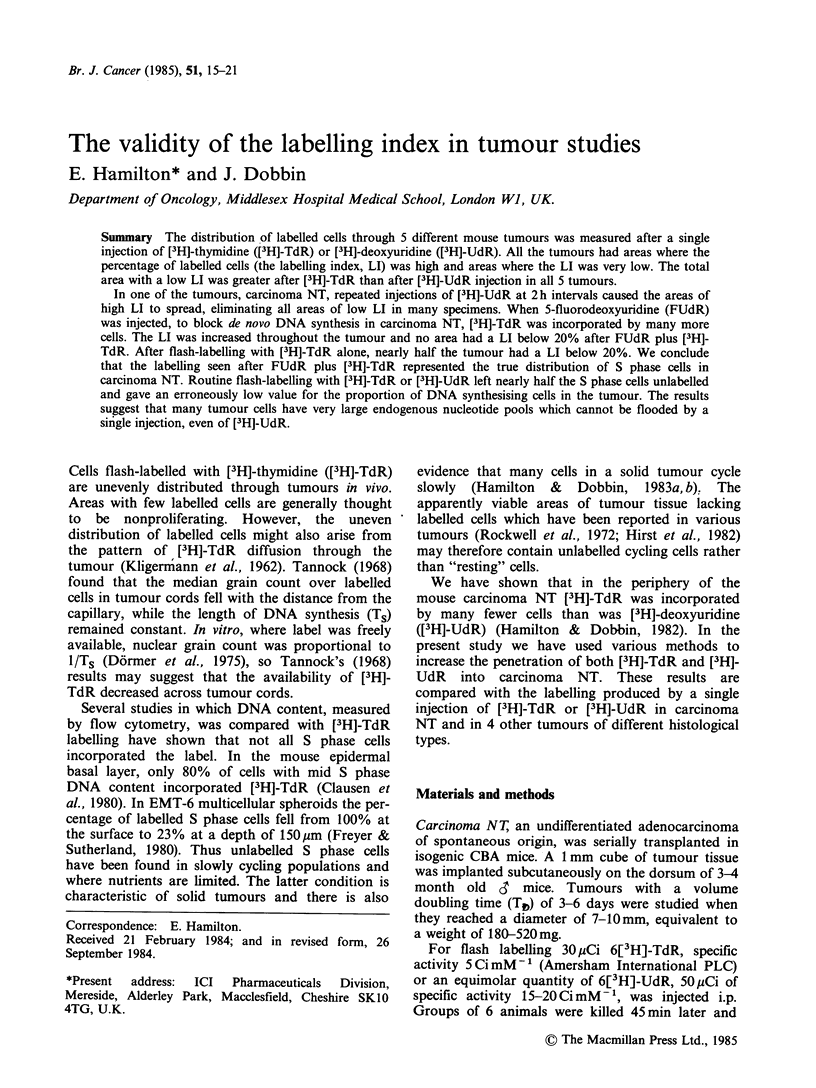

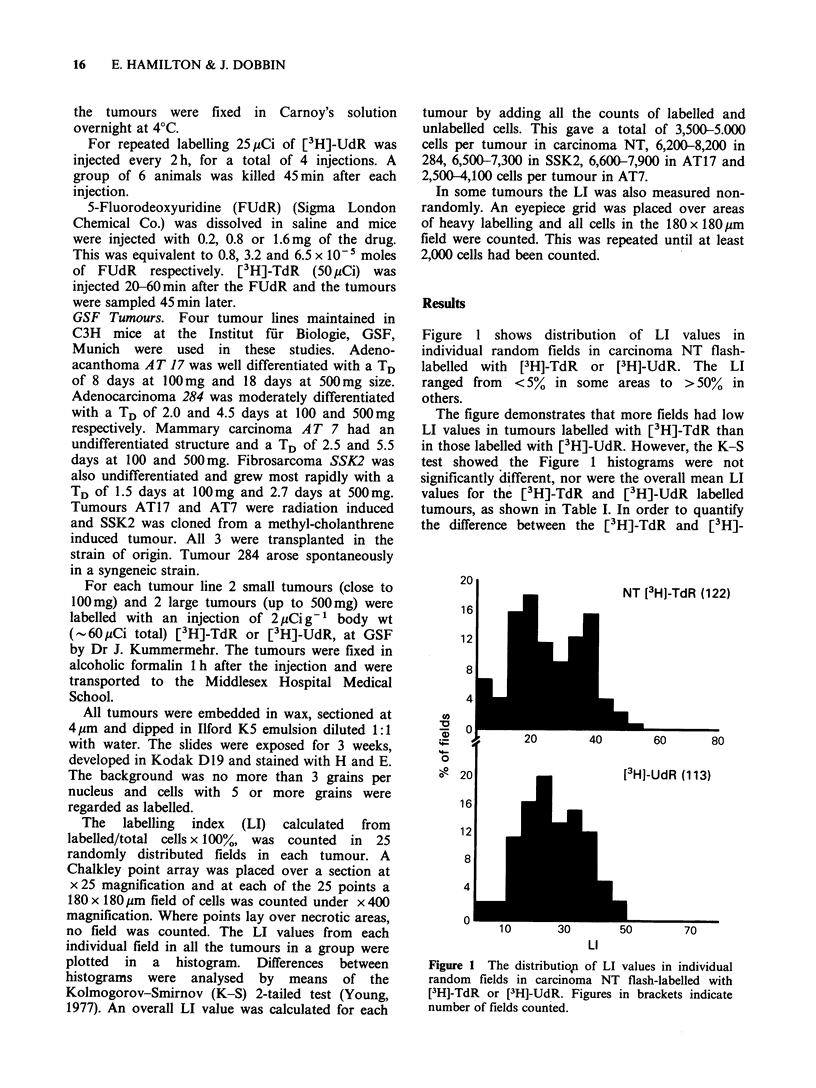

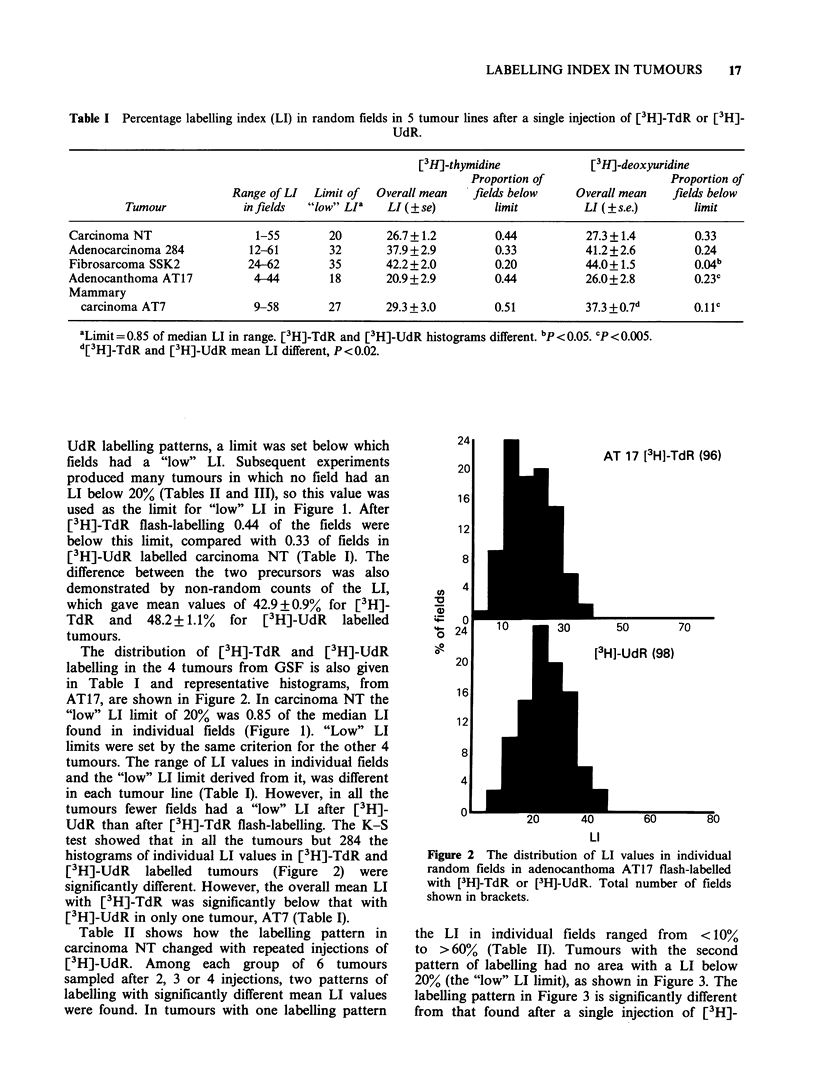

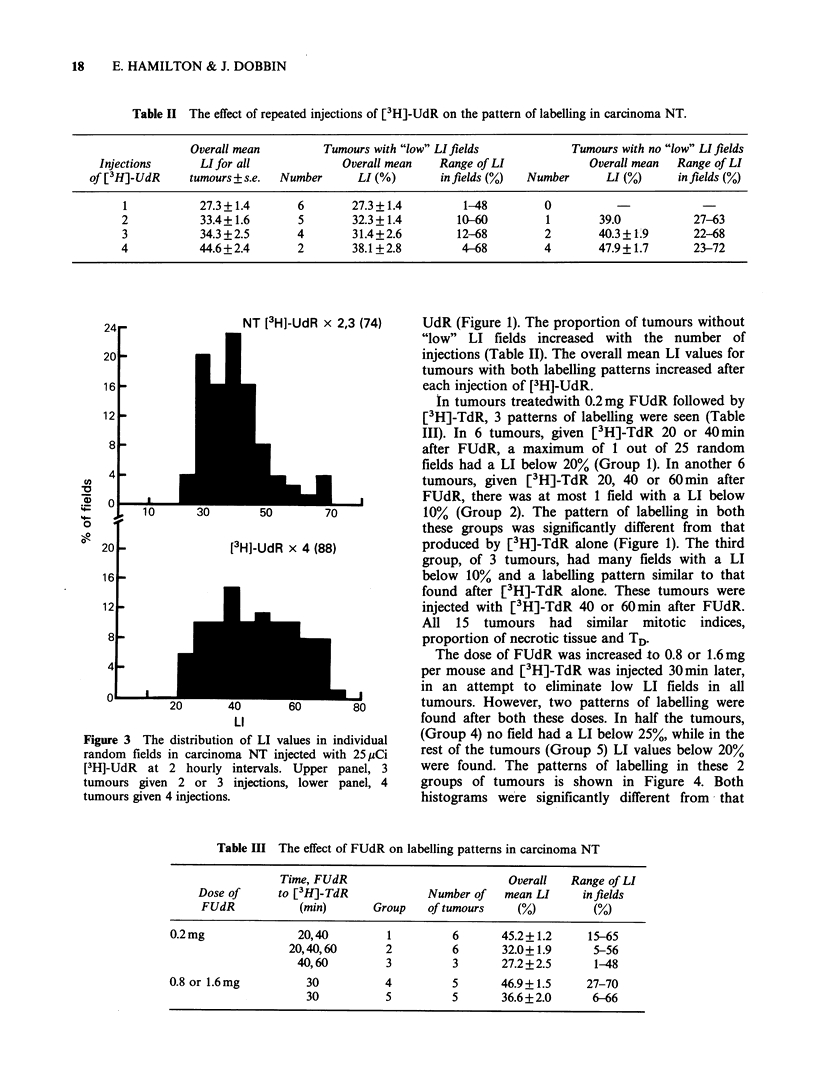

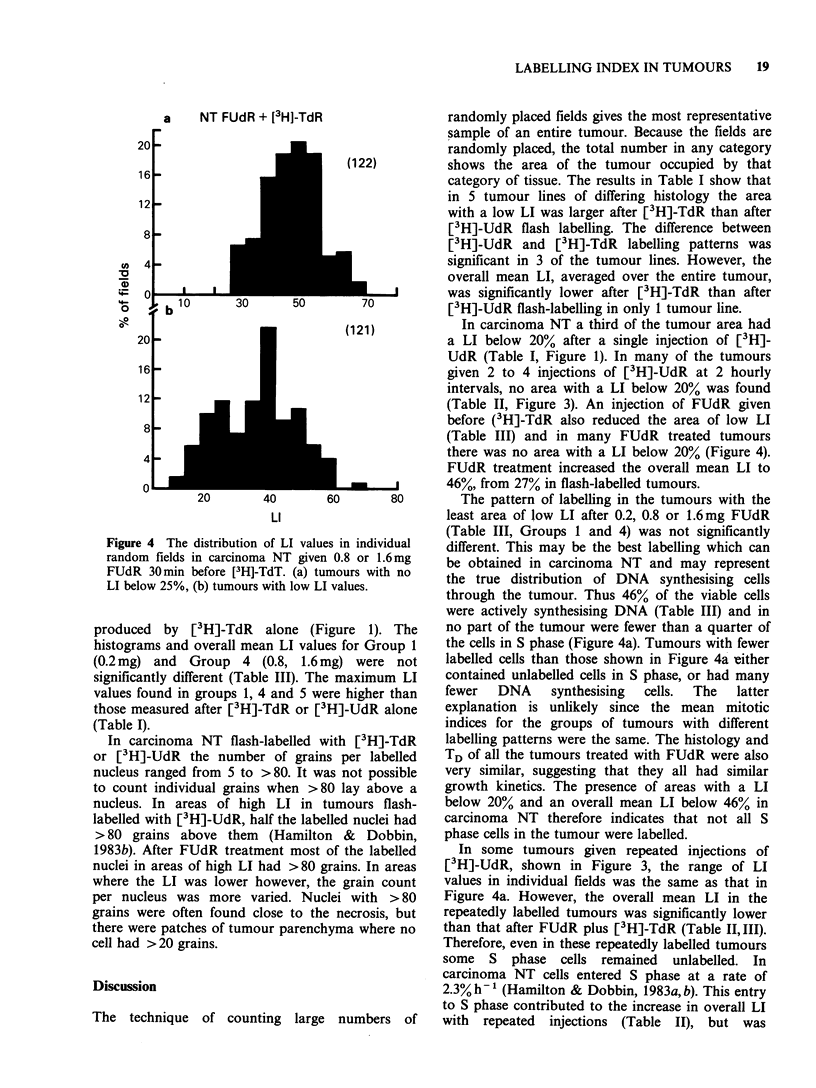

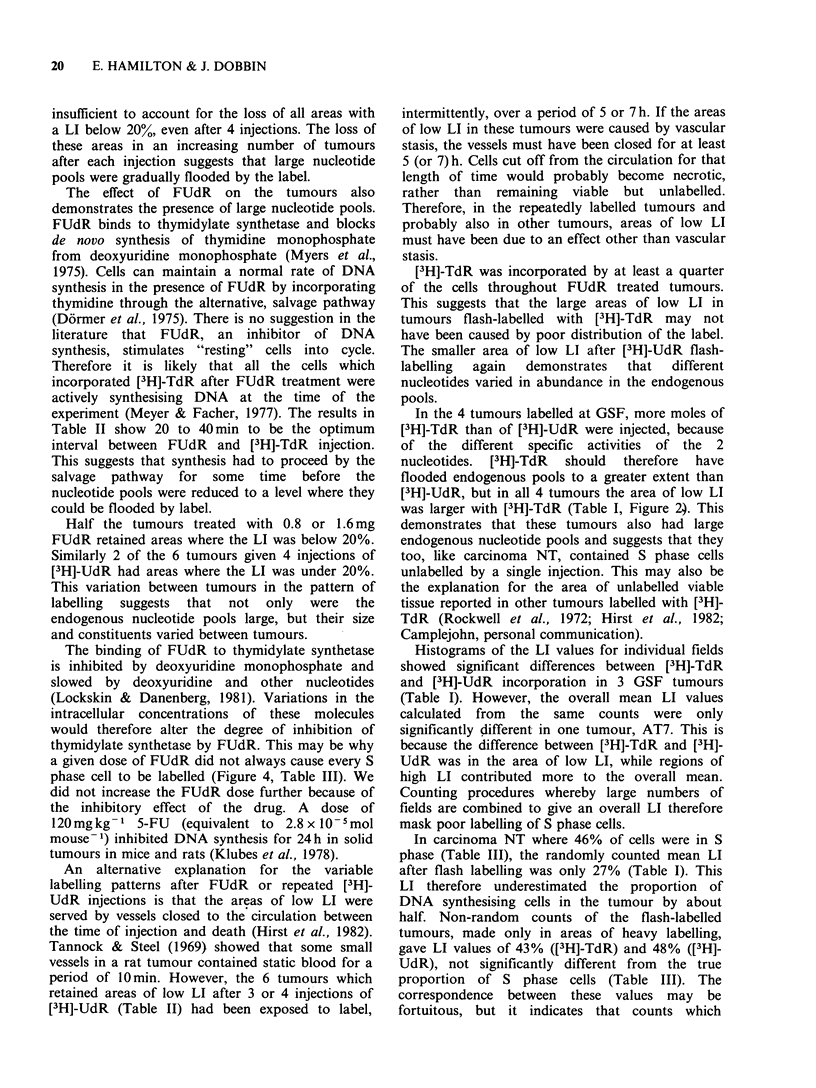

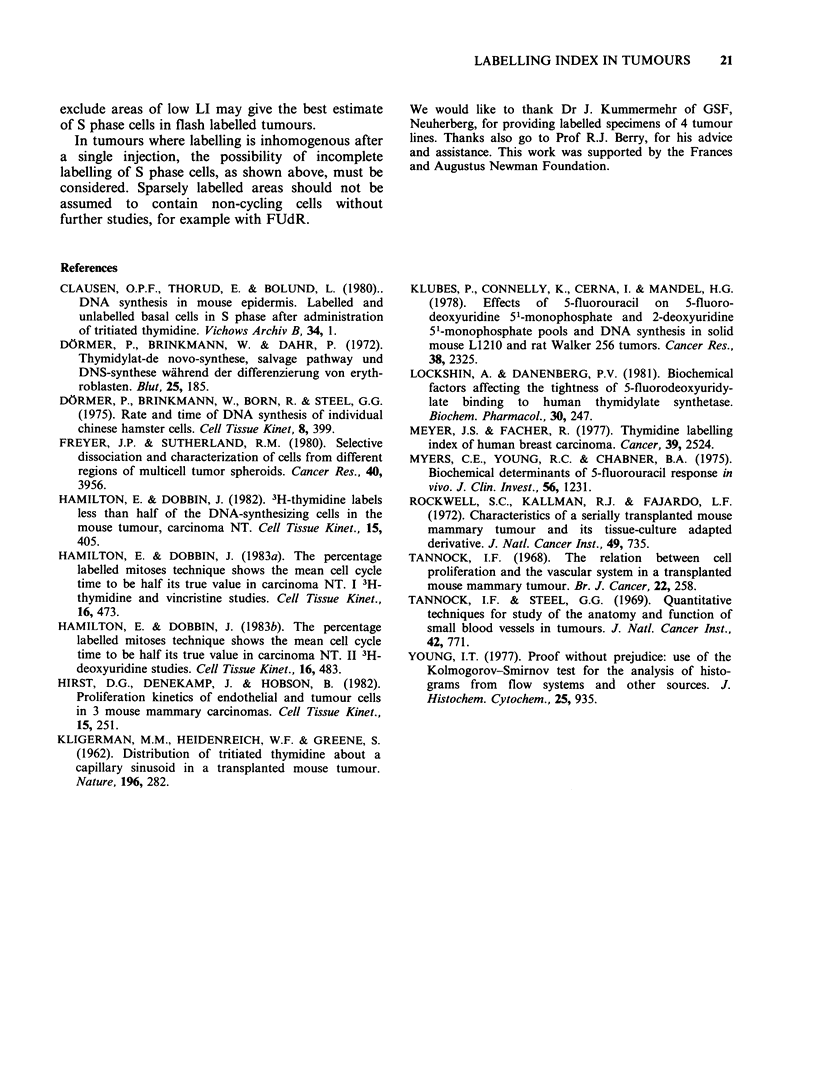


## References

[OCR_00831] Dörmer P., Brinkmann W., Born R., Steel G. G. (1975). Rate and time of DNA synthesis of individual Chinese hamster cells.. Cell Tissue Kinet.

[OCR_00825] Dörmer P., Brinkmann W., Dahr P. (1972). Thymidylat-de novo-Synthese, salvage pathway und DNS-Synthese während der Differenzierung von Erythroblasten.. Blut.

[OCR_00836] Freyer J. P., Sutherland R. M. (1980). Selective dissociation and characterization of cells from different regions of multicell tumor spheroids.. Cancer Res.

[OCR_00848] Hamilton E., Dobbin J. (1983). The percentage labelled mitoses technique shows the mean cell cycle time to be half its true value in Carcinoma NT. I. [3H]thymidine and vincristine studies.. Cell Tissue Kinet.

[OCR_00855] Hamilton E., Dobbin J. (1983). The percentage labelled mitoses technique shows the mean cell cycle time to be half its true value in Carcinoma NT. II. [3H]deoxyuridine studies.. Cell Tissue Kinet.

[OCR_00842] Hamilton E., Dobbin J. (1982). [3H]thymidine labels less than half of the DNA-synthesizing cells in the mouse tumour, carcinoma NT.. Cell Tissue Kinet.

[OCR_00861] Hirst D. G., Denekamp J., Hobson B. (1982). Proliferation kinetics of endothelial and tumour cells in three mouse mammary carcinomas.. Cell Tissue Kinet.

[OCR_00867] KLIGERMAN M. M., HEIDENREICH W. F., GREENE S. (1962). Distribution of tritiated thymidine about a capillary sinusoid in a transplanted mouse tumour.. Nature.

[OCR_00873] Klubes P., Connelly K., Cerna I., Mandel H. G. (1978). Effects of 5-fluorouracil on 5-fluorodeoxyuridine 5'-monophosphate and 2-deoxyuridine 5'-monophosphate pools, and DNA synthesis in solid mouse L1210 and rat Walker 256 tumors.. Cancer Res.

[OCR_00881] Lockshin A., Danenberg P. V. (1981). Biochemical factors affecting the tightness of 5-fluorodeoxyuridylate binding to human thymidylate synthetase.. Biochem Pharmacol.

[OCR_00887] Meyer J. S., Facher R. (1977). Thymidine labeling index of human breast carcinoma. Enhancement of in vitro labeling by 5-fluorouracil and 5-fluoro-2'-deoxyuridine.. Cancer.

[OCR_00891] Myers C. E., Young R. C., Chabner B. A. (1975). Biochemical determinants of 5-fluorouracil response in vivo. The role of deoxyuridylate pool expansion.. J Clin Invest.

[OCR_00896] Rockwell S. C., Kallman R. F., Fajardo L. F. (1972). Characteristics of a serially transplanted mouse mammary tumor and its tissue-culture-adapted derivative.. J Natl Cancer Inst.

[OCR_00907] Tannock I. F., Steel G. G. (1969). Quantitative techniques for study of the anatomy and function of small blood vessels in tumors.. J Natl Cancer Inst.

[OCR_00902] Tannock I. F. (1968). The relation between cell proliferation and the vascular system in a transplanted mouse mammary tumour.. Br J Cancer.

[OCR_00913] Young I. T. (1977). Proof without prejudice: use of the Kolmogorov-Smirnov test for the analysis of histograms from flow systems and other sources.. J Histochem Cytochem.

